# Neuroprotection by Mitochondrial NAD Against Glutamate-Induced Excitotoxicity

**DOI:** 10.3390/cells14080582

**Published:** 2025-04-12

**Authors:** Bruna S. Paiva, Diogo Neves, Diogo Tomé, Filipa J. Costa, Inês C. Bruno, Diogo Trigo, Raquel M. Silva, Ramiro D. Almeida

**Affiliations:** 1 iBiMED—Institute of Biomedicine, Department of Medical Sciences, University of Aveiro, 3810-193 Aveiro, Portugal; brunapaiva@ua.pt (B.S.P.); diogoandreneves92@gmail.com (D.N.); d27diogotome@msn.com (D.T.); fjcosta@ua.pt (F.J.C.); inescbruno@ua.pt (I.C.B.); trigo.diogo@ua.pt (D.T.); 2 CNC—Center for Neuroscience and Cell Biology, University of Coimbra, 3004-504 Coimbra, Portugal; 3 CiBB—Centre for Innovative Biomedicine and Biotechnology, University of Coimbra, 3000-504 Coimbra, Portugal; 4 Center for Interdisciplinary Research in Health, Faculty of Dental Medicine, Universidade Católica Portuguesa, 3504-505 Viseu, Portugal

**Keywords:** excitotoxicity, mitochondria, NAD metabolism, glutamate

## Abstract

Excitotoxicity is a pathological process that occurs in many neurological diseases, such as stroke or epilepsy, and is characterized by the extracellular accumulation of high concentrations of glutamate or other excitatory amino acids (EAAs). Nicotinamide adenine dinucleotide (NAD) depletion is an early event following excitotoxicity in many in vitro and in vivo excitotoxic-related models and contributes to the deregulation of energy homeostasis. However, the interplay between glutamate excitotoxicity and the NAD biosynthetic pathway is not fully understood. To address this question, we used a primary culture of rat cortical neurons and found that an excitotoxic glutamate insult alters the expression of the NAD biosynthetic enzymes. Additionally, using a fluorescent NAD mitochondrial sensor, we observed that glutamate induces a significant decrease in the mitochondrial NAD pool, which was reversed when exogenous NAD was added. We also show that exogenous NAD protects against the glutamate-induced decrease in mitochondrial membrane potential (MMP). Glutamate excitotoxicity changed mitochondrial retrograde transport in neurites, which seems to be reversed by NAD addition. Finally, we show that NAD and NAD precursors protect against glutamate-induced cell death. Together, our results demonstrate that glutamate-induced excitotoxicity acts by compromising the NAD biosynthetic pathway, particularly in the mitochondria. These results also uncover a potential role for mitochondrial NAD as a tool for central nervous system (CNS) regenerative therapies.

## 1. Introduction

L-glutamate is the main excitatory neurotransmitter of the central nervous system (CNS). Glutamate binds to receptors on the postsynaptic neuron, including ionotropic receptors such as N-methyl-D-aspartate receptors (NMDARs), α-amino-3-hydroxy-5-methyl-4-isoxazolepropionic acid receptors (AMPARs), and kainate receptors (KARs), as well as metabotropic glutamate receptors (mGluRs). This interaction facilitates excitatory synaptic transmission and regulates synaptic plasticity processes like long-term potentiation (LTP) and long-term depression (LTD) [1]. However, the excessive release of glutamate and stimulation of its receptors in the mammalian brain leads to excitotoxicity, which is considered the main cause of cell death in acute CNS injuries such as ischemic stroke [2,3]. This overactivation, typically involving the NMDAR, results in massive calcium diffusion through Ca channels, transported by Na/Ca exchangers or Ca-ATPases, that triggers several biochemical cascades, which lead to synaptic damage and neuronal death, and, as a consequence, to altered glutamatergic neurotransmission [4,5]. Glutamate excitotoxicity leads to oxidative stress and mitochondrial impairment, and, as a result, to a failure in energy metabolism, culminating in cell demise [6].

Nicotinamide adenine dinucleotide (NAD) depletion is an early event after an excitotoxic insult [7,8,9]. Both oxidative stress and calcium overload contribute to the activation of poly(ADP-ribose)-1 (PARP-1), a NAD-dependent enzyme involved in DNA repair. PARP-1 activation results in NAD depletion, which is exacerbated by impaired mitochondrial function [1]. NAD is essential for energy production and cell metabolism as a coenzyme for oxidation-reduction reactions that result in ATP synthesis. NAD biosynthesis in mammals is supported by several precursors and occurs through four main pathways that use distinct sets of enzymes. The de novo pathway utilizes tryptophan as a precursor, while salvage pathways rely on nicotinamide (NAM), nicotinic acid (NA), and nicotinamide riboside (NR). NAM and NA are converted into their respective mononucleotides by nicotinamide phosphoribosyltransferase (NAMPT) and nicotinic acid phosphoribosyltransferase (NAPRT), respectively. The mononucleotides NMN and NAMN are then transformed into NAD or its precursor NAAD by NMN adenyltransferases (NMNAT1–3). NMNAT1 is found in the nucleus; NMNAT2 localizes to the Golgi and cytoplasm; while NMNAT3 is present in mitochondria but can also be found in the cytoplasm. NAAD can subsequently be converted into NAD by NAD synthetase 1 (NADS). Additionally, NMN can also be produced through the phosphorylation of nicotinamide riboside (NR) by nicotinamide riboside kinases 1 and 2 (NMRK1/2) [1]. In the context of excitotoxicity, there is evidence that NAD, NAD precursors, and NAD biosynthetic enzymes can be neuroprotective following an excitotoxic insult. In neuronal primary cultures exposed to glutamate, NR and NAD protect against excitotoxicity-induced axonal degeneration [10], while NAD alone improves mitochondrial biogenesis and function [11]. Furthermore, the overexpression of the NAM-associated rate-limiting enzyme NAMPT was linked with the suppression of both mitochondrial fragmentation and loss of mitochondrial DNA in neurons [12].

In this work, we used cortical rat neurons to further elucidate the alterations in NAD metabolism in response to excitotoxic glutamate damage. We show that the NAD biosynthesis enzymes display an altered expression profile following excitotoxic glutamate injury and that the administration of either NAD or the NAD precursors, NAM and NA, improves cell survival and metabolic function upon glutamate exposure. Glutamate excitotoxicity altered velocity and displacement in neurons. We also found that NAD decreases after glutamate stimulation and that treatment with exogenous mitochondrial NAD is able to rescue the glutamate-induced loss of mitochondrial NAD levels and the decrease in MMP. Overall, these results show that neuronal protection conferred by NAD metabolism during excitotoxicity is mediated, at least in part, by the mitochondrial NAD pool.

## 2. Material and Methods

### 2.1. Primary Neuronal Cultures

Wistar-Han rats were housed and maintained in a controlled environment at 22–24 °C with 55% humidity, on a 12 h light/dark cycle, and fed with regular rodent chow and water ad libitum. Primary cultures of rat cortical neurons were prepared as previously described [13]. Briefly, cortices from E17–E18 Wistar rat embryos were incubated with trypsin (0.045%;Gibco, Thermo Fisher Corporation, Waltham, MA, USA) and deoxyribonuclease (0.01% *v/v*; Sigma-Aldrich, St. Louis, MO, USA) in Hank’s balanced salt solution (HBSS) (5.36 mM KCl, 0.44 mM KH_2_PO_4_, 137 mM NaCl, 4.16 mM NaHCO_3_, 0.34 mM Na_2_HPO_4_·2H_2_O, 5 mM glucose, 1 mM sodium pyruvate, 10 mM HEPES, 0.001% phenol red, pH 7.2) for 15 min at 37 °C. Afterwards, Hank’s solution with trypsin was removed, and the tissue was washed once with plating medium (MEM + 0.35% glucose + 10% FBS), in order to stop the trypsin action. Then, the tissue was mechanically dissociated and cell density was determined. Cells were plated on glass coverslips, previously coated with 0.1 mg/mL of poly-D-lysine (PDL) (Millipore, Burlington, MA, USA) at a density of 45 × 10^3^ cells/cm^2^ and maintained in culture medium [Neurobasal medium (Gibco) supplemented with 2% B27 (Gibco, Thermo Fisher Corporation, Waltham, MA, USA), 0.5 mM glutamine (Gibco, Thermo Fisher Corporation, Waltham, MA, USA), and 1:400 penicillin/streptomycin (Gibco, Thermo Fisher Corporation, Waltham, MA, USA)]. For live cell imaging, 35 mm dishes were coated with 0.1 mg/mL of PDL, washed three times with water, and coated with 2 μg/mL of laminin. Cells were plated at 30,000 cells/cm^2^. Cells were maintained at 37 °C in a humidified incubator under an atmosphere of 95% air and 5% CO_2_. After 3/4 days in vitro (DIV), the mitotic inhibitor 5-fluoro-2′-deoxyuridine thymidylate synthase inhibitor (5-FDU) (10 μM; Sigma Aldrich, St. Louis, MO, USAwas added to the cultures to reduce contamination with glial cells. Neurons were grown in vitro until DIV 7/8 before used in the different experimental setups.

### 2.2. Cell Viability Assay

To evaluate the neuroprotective role of the NAD biosynthetic pathways in glutamate-induced excitotoxicity, cells were pre-incubated with 3 mM nicotinic acid (NA) for 3 h, 15 mM nicotinamide (NAM), or 5 mM NAD for 1 h before glutamate stimulation at DIV8. In one-third of their conditioned medium, cells were then challenged with 50 µM glutamate (Sigma) for 15 min. Afterwards, the medium with glutamate was replaced by a glutamate-free conditioned medium that had been previously saved. The viability of the cultures was evaluated 6 h later. Then, the cells were washed three times with phosphate-buffered saline (PBS) [137 mM NaCl, 2.7 mM KCl, 10 mM Na_2_HPO_4_, 1.8 mM KH_2_PO_4_, pH 7.4] and fixed in 4% paraformaldehyde (in PBS with 4% sucrose) for 10 min at room temperature. Following three washes with PBS to remove the paraformaldehyde solution, cells were incubated with the fluorescent dye Hoechst 33,342 (2 μg/mL) diluted in PBS for 10 min at room temperature. Finally, cells were washed twice in PBS, and the coverslips were then mounted using ProLong mounting medium (Molecular probes, Thermo Fisher Corporation, Waltham, MA, USA). The preparations were cured overnight at 4 °C, protected from light, and sealed with nail polish. Fluorescent images of fixed cells were obtained with a Plan-Apochromat 40× objective in a Zeiss Observed Z.1 microscope (Carl Zeiss Microscopy, Jena, Germany), equipped with an AxioCam HRm camera, (Carl Zeiss Microscopy, Jena, Germany) and the Zen Blue 2011 software (Carl Zeiss Microscopy, Jena, Germany). Cell counting was performed based on stained nuclei in 15–20 randomly picked images from different locations within each coverslip. The percentage of condensed nuclei was determined to evaluate neuronal cell death under each experimental condition.

### 2.3. Protein Extraction and Immunoblotting

For total cell extract, cortical neurons were lysed in RIPA buffer (150 mM NaCl, 1% NP40, 0.5% sodium deoxycholate, 0.1% SDS, 50 mM Tris pH 8.0) with protease and phosphatase inhibitors (Roche, Basel, Switzerland), followed by total protein quantification using the Pierce™ BCA Protein Assay Kit (Thermo Fisher Scientific, Waltham, MA, USA). Protein samples (12 µg) were separated by SDS-PAGE gels, transferred to 0.45 μm nitrocellulose membranes (GE Health Care, Boston, MA, USAand blocked with 5% low-fat milk in TBS-0.5% Tween. The following primary antibodies were used for immunoblotting: anti-NAMPT (HPA047776,Sigma Aldrich, St. Louis, MO, USA, 1:2000 dilution), anti-NAPRT (HPA024017,Sigma Aldrich,, St. Louis, MO, USA, 1:2000 dilution), and anti-β-Actin (NB600-501, Novus Biologicals, Centennial, CO, USA, 1:10,000 dilution). IgG HRP-linked secondary antibodies were anti-rabbit (7074S, Cell Signaling Technology, Leiden, The Netherlands, 1:5000 dilution) and anti-mouse (7076S, Cell Signaling, 1:5000 dilution). Membranes were subjected to ECL detection (GE Healthcare, Buckinghamshire, UK), and images were acquired using Chemidoc (Bio-Rad, Hercules, CA, USA).

### 2.4. RNA Extraction, cDNA Conversion, and RT-PCR

Cortical neurons were lysed with TRIZOL at room temperature, and RNA isolation and purification were performed with Direct-zol™ RNA MiniPrep (Zymo Research, Irvine, CA, USA). One microgram of purified RNA was reverse transcribed using a SensiFast cDNA synthesis kit (Meridian Bioscience, Memphis, TN, USA). Gene expression of *NAMPT*, *NMNAT1*, *NMNAT2*, *NMNAT3*, and *18s* rDNA was assessed by Reverse Transcription Polymerase Chain Reaction (RT-PCR) using the QIAGEN Multiplex PCR kit (QIAGEN, Milan, Italy). The PCR settings were as follows: initial denaturation step for 15 min at 95 °C, followed by 35 cycles of a denaturation (30 s, 94 °C)—annealing (30 s, 57 °C)—extension (30 s, 72 °C) step, and a final extension for 10 min at 72 °C. Forward and reverse primers with respective lengths and annealing temperatures are described in Supplementary Table S1. The samples were amplified in a MyCycler™ thermocycler (Bio-Rad, Hercules, CA, USA). The RT-PCR end products were resolved by 1.5% agarose gel electrophoresis, and changes in gene expression were evaluated using the Gel Doc XR+ (Bio-Rad, Hercules, CA, USA).

### 2.5. Transient Transfection with NAD Biosensors

Cortical neurons were transiently transfected at DIV3/4 with 3 µg per 45 × 10^3^ cells/cm^2^ of mitochondrial NAD and cpVenus control sensors, which emit fluorescence at 488 nm, using a calcium phosphate co-precipitation protocol, as described in [13]. Cells at a density of 45 × 10^3^ cells/cm^2^ were incubated with 10 mM Kynurenic acid (KA) for 20 min. Then, a mixture containing TE (1 mM Tris-HCl, 250 mM EDTA pH = 7.15), DNA, and 2.5 µL of 250 mM CaCl_2_ in a total volume of 25 µL was added to 25 µL of HEBS solution (274 mM NaCl, 10 mM KCl, 1.4 mM Na_2_HPO_4_, 15 mM D-glucose, 42 mM HEPES, pH 7.05). The precipitates were immediately added to the cells containing KA and incubated for 2 h. After this period, the transfection was stopped by adding HCl to a conditioned medium containing KA (until the solution turned yellow) for 20 min. Cells were then washed 3 times with fresh cell medium and allowed to incubate for 48 h with the sensors. At DIV 5, cells were treated with 5 mM NAD for 1 h before glutamate stimulation. Cells were then challenged with 50 µM glutamate (Sigma) for 15 min and analyzed by immunocytochemistry 24 h later as described in the following section.

### 2.6. Immunocytochemistry and Cell Imaging Analysis

Transfected neurons were fixed in 4% paraformaldehyde for 15 min, and permeabilized with 0.2% Triton X-100 in PBS for 10 min. Afterwards, neurons were washed with PBS, blocked with 3% BSA in PBS for 1 h, followed by incubation with the primary antibodies anti-βIII-tubulin (T8578, Sigma Aldrich, 1:500 dilution) and anti-TOM20 (11802-1-AP, ProteinTech, Manchester, UK, 1:250 dilution) for 2 h. Next, cells were incubated with Alexa-Fluor anti-rabbit 568 secondary antibody (1:1000 dilution) for 1 h. Finally, the coverslips were mounted on a microscope slide with DAPI containing VECTASHIELD^®^ mounting media (Vector Laboratories, Newark, NJ, USA). Samples were visualized under a Zeiss LSM 880 confocal laser scanning microscope with an Airyscan (Carl Zeiss Microscopy, Jena, Germany) and a 63× oil immersion objective. The sum of the intensities of 10 z-stacks was used in ImageJ (version 1.54g, National Institutes of Health, Bethesda, MD, USA) to create a Z-projection for each image, and then the corrected total cell fluorescence (CTCF) of the NAD sensors was quantified by subtracting the fluorescence of the adjacent background. To obtain relative CTCF levels, glutamate and glutamate plus NAD conditions were normalized to the control condition. For each experiment, a total of 25 cells were quantified.

### 2.7. Metabolic Assay

Metabolic activity was evaluated at DIV 8 using the Alamar Blue assay. Cells were pre-incubated with 3 mM nicotinic acid (NA) for 3 h, or 15 mM nicotinamide (NAM), or 5 mM NAD for 1 h before glutamate stimulation. Cells were then challenged with 50 µM glutamate (Sigma-Aldrich, St. Louis, MO, USA) for 15 min in one-third of their conditioned medium. Afterward, the medium with glutamate was replaced by glutamate-free conditioned medium that had been previously saved. Six hours after glutamate treatment, the culture medium was removed, and 5 µg/mL Alamar Blue reagent (Bio-rad) diluted in fresh Neurobasal medium containing glutamine, penicillin/streptomycin, and B27 was added to the cells for 45 min at 37 °C. Afterward, absorbance was read at 570 nm and 600 nm on a Microplate Reader SpectraMax iD3 (Molecular Devices, San Jose, CA, USA).

### 2.8. Mitochondrial Morphology and Dynamic Assay

To better understand how glutamate excitotoxicity affects mitochondrial morphology and dynamics, we used tetramethylrhodamine, methyl ester (TMRM, T668; Thermo Fisher Scientific, Waltham, MA, USA). TMRM is a cell-permeant fluorescent dye, sequestered by active mitochondria. Cells were plated in 35 mm dishes (Ibidi, Gräfelfing, Germany) at a concentration of 30,000 cells/cm^2^. At DIV 8, cells were loaded with 20 nM TMRM for 30 min. Live-cell timelapse micrographs (200 frames, 2-s intervals) were recorded from five imaging fields per preparation, using a confocal microscope (LSM 510 Meta, Carl Zeiss Microscopy, Jena, Germany). Images were taken with the cells in neurobasal medium at 37 °C. These images were later analyzed using a routine optimization of the MATLAB (version R2022a) algorithm mitometer [14,15], which provided mitochondrial parameters, such as movement (displacement and velocity). Due to the size of generated files and processing requirements, the MATLAB analysis was restricted to the first 50 frames. the Fiji distribution of ImageJ software, (version 1.54g, National Institutes of Health, MD, USA) was used to generate kymographs of all 200 captured frames of mitochondrial movement in the neurites and assess movement directionality, selecting one continuous neurite (average length 75 µm) per imaging field.

### 2.9. Mitochondrial Membrane Potential Assay

Rat embryonic cortical neurons were incubated with JC-10 (TCI America, Montgomeryville, OR, USA), at a dilution of 1:1000, to evaluate the mitochondrial membrane potential. At DIV 7, the cells were pre-incubated with 5 mM NAD for 1 h before glutamate stimulation. Cells were then challenged with 50 µM glutamate (Sigma) for 15 min in one-third of their conditioned medium. Afterwards, the medium with glutamate was replaced by glutamate-free conditioned medium that had been previously saved. Twenty-four hours after glutamate treatment, the culture medium was removed and 10 μM JC-10 probe resuspended in Krebs buffer (145 mM NaCl, 5 mM KCl, 10 mM HEPES, 1 mM MgCl_2_, 1 mM CaCl2, 5.6 mM glucose, and pH 7.4/NaOH) was added to each well and incubated at 37 °C, 5% CO_2_ for 30 min and fluorescence intensities (Ex/Em = 490/525 nm and Ex/Em = 540/590 nm) were acquired in a plate reader (Infinite m200, Tecan Life Sciences, Männedorf, Switzerland). As a positive control, 30 µM FCCP was added to the cells for 2 h before JC-10 measurement.

### 2.10. Statistical Analysis

Results are presented as averaged values ± SEM of at least three independent experiments. Graphs and statistical analysis were performed in GraphPad Prism 8 software. Statistical significance was assessed by unpaired *t*-student test, one-way ANOVA followed by Bonferroni’s multiple comparison test or Sidak’s multiple comparison test. Values of *p* < 0.05 were considered statistically significant.

## 3. Results

### 3.1. Expression of NAD Biosynthetic Pathway Enzymes Is Altered in Response to Glutamate-Induced Excitotoxicity

**Figure 1:**
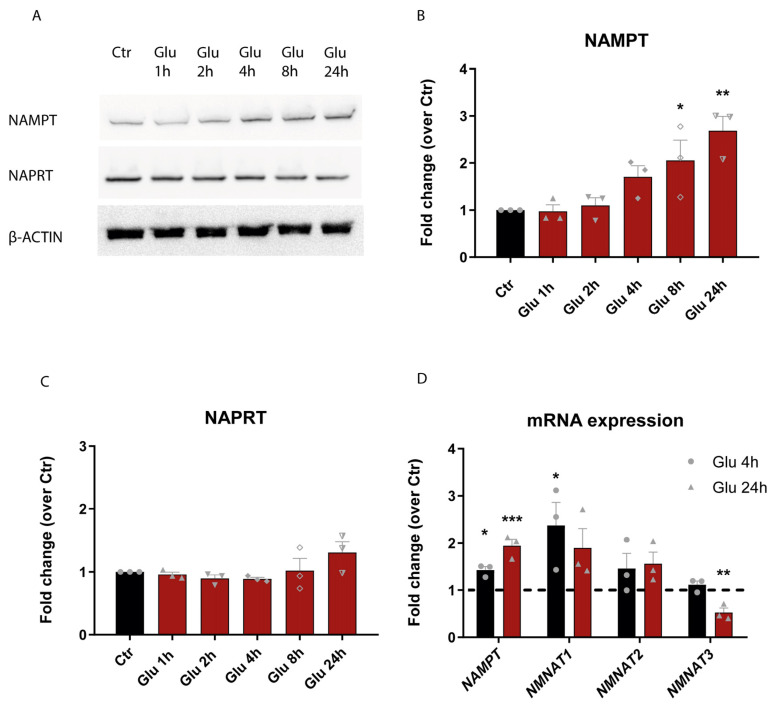
Alterations in the expression of NAD biosynthetic enzymes following glutamate-induced excitotoxicity. (**A**–**C**) Representative images and quantitative data of NAMPT and NAPRT enzymes. Analysis of NAMPT and NAPRT protein expression. Rat cortical neurons were exposed to 50 µM glutamate for 15 min and protein levels were determined at the indicated times by Western blot. β-Actin was used as a loading control (**A**). We observed an increase in NAMPT and no alterations in NAPRT protein expression after glutamate stimulation (**B**,**C**). (**D**) Quantitative data of NAMPT and NNMAT enzymes mRNA expression. Evaluation of NAMPT, NMNAT1, NMNAT2, and NMNAT3 mRNA expression. Rat cortical neurons were exposed to 50 µM glutamate for 15 min and mRNA expression was analyzed after 4 h and 24 h. RT-PCR results indicated an increase in gene expression levels for NAMPT and NMNAT1 after glutamate stimulation and a decrease in NMNAT3 levels following 24 h of glutamate stimulation. 18s rDNA was used as an internal control gene. The dashed line represents normalization to the control condition for each gene. Bars represent the mean ± SEM of 3 independent experiments; * represents *p* < 0.05; ** represents *p* < 0.01 and *** represents *p* < 0.001 by ANOVA followed by Dunnett’s multiple comparisons test when compared to control.

NAD depletion is an early event following glutamate-induced excitotoxicity; however, which NAD biosynthetic enzymes respond to glutamate-induced damage is unknown. To address this question, we evaluated the protein expression of NAMPT, the rate-limiting enzyme of the NAM route for NAD biosynthesis in mammals, and NAPRT, the main enzyme of the route from the precursor NA. Rat cortical neurons were exposed to 50 µM glutamate for 15 min, and protein levels were analyzed 4 h, 8 h, or 24 h later by Western blot. We observed a significant increase in NAMPT protein expression (Figure 1A,B) but no alterations in NAPRT protein levels (Figure 1A,C). Therefore, NAMPT-mediated NAD biosynthesis seems to be the main responder after glutamate-induced excitotoxicity. To further explore this observation, we considered the last step of NAD formation in the NAM route, which involves nicotinamide mononucleotide adenylyltransferases (NMNATs), which have three compartmentalized isoforms. NMNAT1 generates NAD in the nucleoplasm, NMNAT2 in the cytosol, and NMNAT3 in mitochondria. Rat cortical neurons were exposed to 50 µM glutamate for 15 min, and mRNA expression was analyzed 4 h or 24 h later by RT-PCR. The results indicate an increase in NAMPT, NMNAT1, and NMNAT2 gene expression levels following 4 h and 24 h of glutamate exposure. Interestingly, NMNAT3 levels decreased after 24 h of glutamate exposure (Figure 1D).

### 3.2. Mitochondrial NAD Levels Decrease Following Glutamate-Induced Excitotoxicity

**Figure 2:**
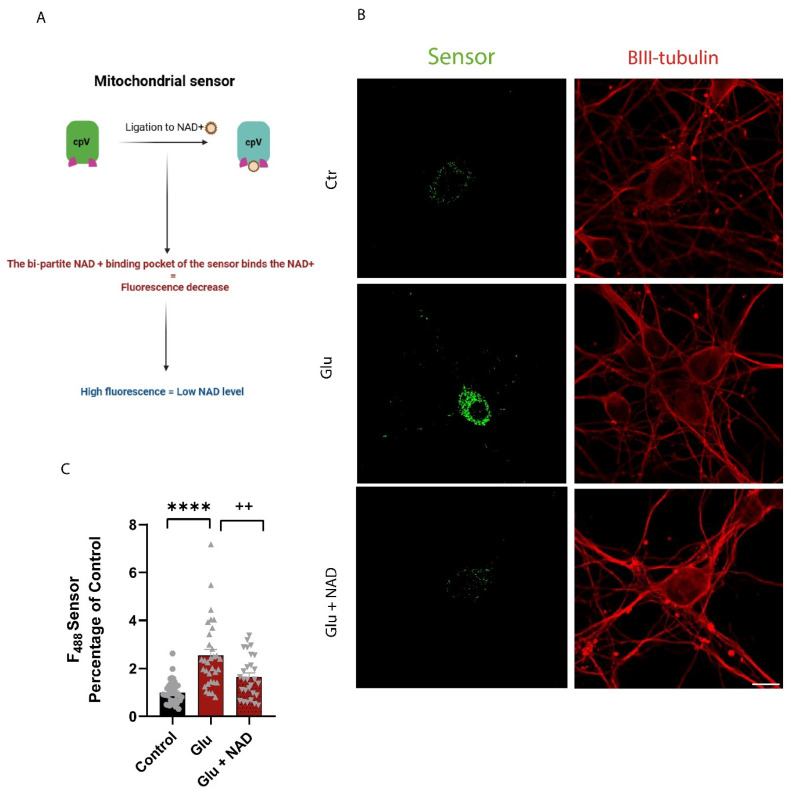
Exogenous NAD^+^ rescues the decrease in mitochondrial NAD^+^ following glutamate-induced excitotoxicity. (**A**) Schematic depiction illustrating the operational mechanism of the mitochondrial sensor. (**B**) Representative images of mitochondrial NAD sensor fluorescence. Sum intensity projections of Z-stacks from cortical neurons transfected with 3 µM mitochondrial NAD sensor (green) and immunolabeled for the neuronal marker βIII-tubulin (red). Sum intensity projections of Z-stacks were assembled to generate a single image. An increase in cell fluorescence represents a decrease in free NAD in the mitochondria. Scale bar: 10 µm. (**C**) Quantitative data of mitochondrial NAD sensor fluorescence. Relative corrected total cell fluorescence was quantified in cortical neurons exposed to glutamate in the presence or absence of NAD. Glutamate (Glu) induces an increase in mitochondrial NAD sensor fluorescence intensity, which is reversed by 5 mM NAD^+^ addition prior to Glu, indicating an increase in free NAD^+^ in the mitochondria. Control (circles); Glutamate (triangles); Glutamate + NAD (upside down triangle) Bars represent the mean ± SEM of at least 30 neurons randomly selected from 3 independent experiments. Statistical significance was assessed by ANOVA followed by Sidak’s multiple comparison test. **** represents *p* < 0.0001 when compared to control. ++ represents *p* < 0.01 when compared to glutamate.

Considering the results obtained for the mitochondrial *NMNAT3*, we took advantage of NAD biosensors capable of measuring free NAD levels within subcellular localizations. These sensors contain a circularly permuted Venus fluorescent protein (cpVenus) and an NAD binding domain responsible for the ligation of NAD and not other metabolites such as NADH or NADP(H) [16,17]. The mitochondrial NAD sensor used in this study shows an inverted correlation with mitochondrial NAD levels. In short, lower fluorescence intensity represents a higher amount of mitochondrial NAD (Figure 2A) [16]. To validate the mitochondrial NAD sensor (mSensor), we transfected cortical neurons with the mSensor. Next, we immunolabeled neurons using an antibody against TOM20, an outer mitochondrial membrane protein, and a widely used mitochondrial marker. The results show co-localization between TOM20 and mSensor (Figure S1), indicating specificity of NAD sensor location.

We next asked whether glutamate impacts the levels of mitochondrial NAD. Cortical neurons were transfected with the mSensor and exposed to 50 µM glutamate for 15 min, and 24 h later, cells were fixed and stained against βIIITub, a neuronal marker. The fluorescence of cpVenus was analyzed in the cell body by confocal microscopy using βIIITub staining (Figure 2B). We observed an increase in mSensor fluorescence in cells exposed to glutamate compared to control cells, indicating a depletion of the mitochondrial NAD pool. Interestingly, NAD addition prior to glutamate resulted in a decrease in sensor fluorescence compared to glutamate alone (Figure 2C), indicating that the levels of mitochondrial NAD can be maintained under excitotoxic conditions if exogenous NAD is supplied. To rule out the possibility that this observation results from a non-specific effect of glutamate on the stability of the fluorescence protein, a cpVenus sensor control lacking the NAD binding domain was used in parallel. No alterations in the fluorescence of the control sensor were observed under all the conditions tested (Figure S2), validating the results obtained with the mSensor. Overall, the results presented here show that exogenous NAD rescues the decrease in mitochondrial NAD levels following glutamate-induced excitotoxicity.

### 3.3. NAD Rescues the Decrease in MMP Promoted by Glutamate-Induced Excitotoxicity

**Figure 3:**
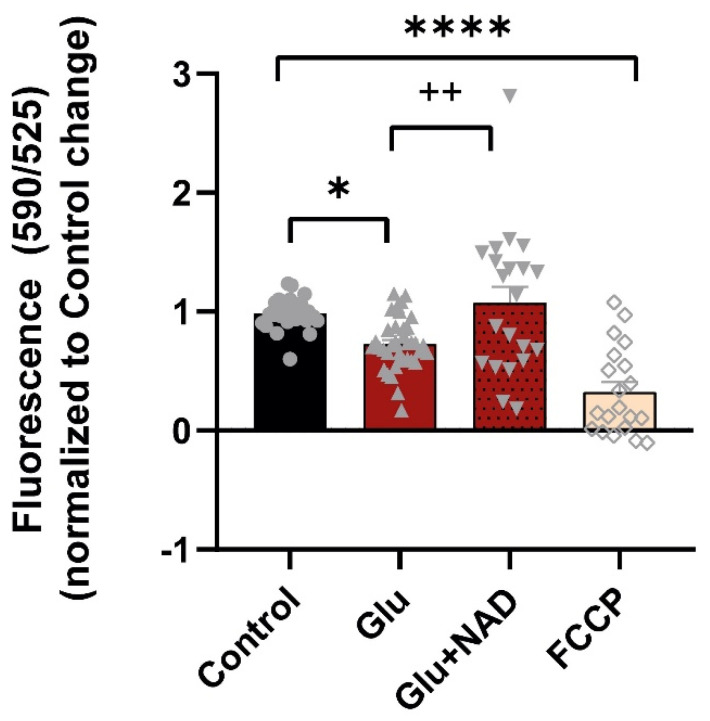
NAD reverts the decrease in MMP induced by glutamate excitotoxicity. Mitochondrial membrane potential (MMP) was measured in cortical neurons with the JC-10 probe. Exposure to 50 µM glutamate (Glu) results in a loss of MMP that is prevented by 5 mM NAD+ addition. Control (circles); Glutamate (triangles); Glutamate + NA (upside down triangle); FCCP (empty diamondsBars represent the mean ± SEM from 6 independent experiments. Statistical significance was assessed by ANOVA followed by Sidak’s multiple comparison test. * represents *p* < 0.05; **** represents *p* < 0.0001 when compared to control. ++ represents *p* < 0.01 when compared to glutamate.

To evaluate the impact of glutamate excitotoxicity on mitochondrial membrane potential, cortical neurons were incubated with JC-10, a mitochondrial membrane potential sensor. This fluorescent dye is used to assess mitochondrial membrane potential (ΔΨm). The red (λex = 540 nm/λem = 590 nm)/green fluorescence (λex = 490 nm/λem = 525 nm) intensity ratio provides a quantitative measure of mitochondrial health. Cortical neurons were exposed to glutamate with or without NAD, and the fluorescence intensity ratio was quantified. Glutamate exposure reduced MMP to 72.7% when compared to the control. Pre-incubation with NAD prior to glutamate insult was able to rescue MMP to 107.5%. FCCP was used as a positive control and induced a decrease in MMP to 32.6% when compared to the control (Figure 3). The results indicate that NAD is able to prevent the mitochondrial depolarization induced by glutamate.

### 3.4. Glutamate Excitotoxicity Alters Mitochondrial Dynamics in Cortical Neurons

**Figure 4:**
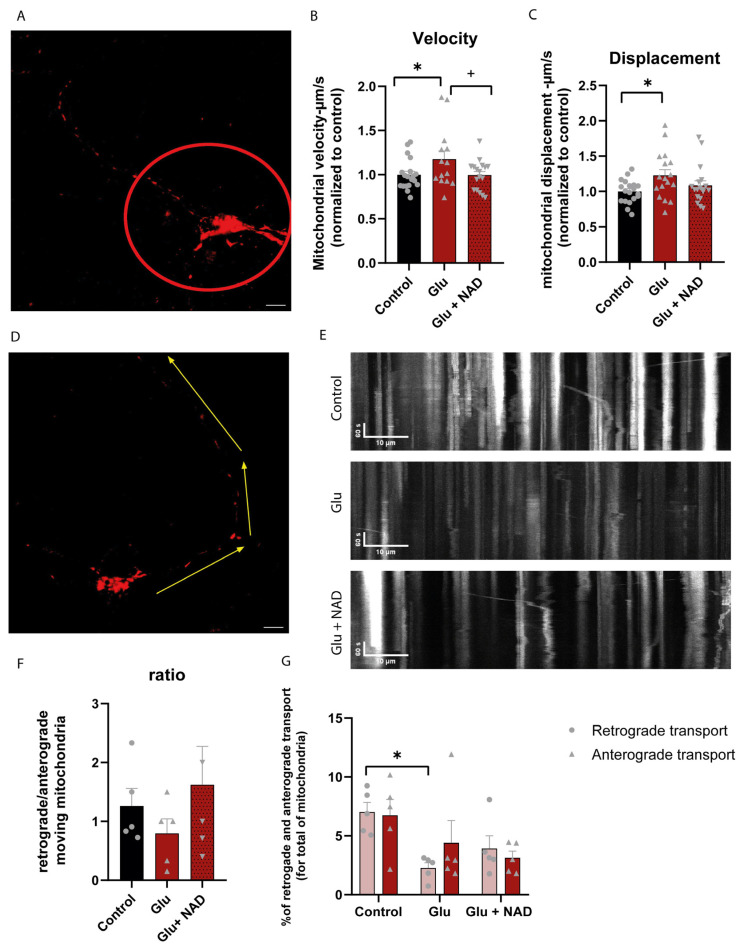
Glutamate excitotoxicity promotes mitochondrial anterograde movement in neurites. (**A**–**C**) Analysis of mitochondrial velocity and displacement in the neurites of cortical neurons. Neurons were exposed to 50 µM glutamate for 15 min in the presence or absence of 5 mM NAD, stained with TMRM, and live imaged by confocal microscopy after 24 h. The mitometer MATLAB algorithm was used to quantify mitochondrial movement in the neurites (i.e., area outside the red circle in (**A**)). Scale bar: 10 µm. Glutamate increased mitochondrial velocity and displacement (**B**,**C**), which were reversed by the presence of NAD. Bars represent the mean ± SEM of 4 independent experiments. * represents *p* < 0.05 when compared to control. + represents *p* < 0.05 when compared to control. Statistical analysis was conducted with ANOVA test (Sidak’s multiple comparison test). (**D**–**G**) Quantitative data of mitochondrial movement in neurites. FIJI software was used to generate kymographs of mitochondria in neurites. Neurite selection was made from the cellular body to the end of the neurite (**D**). Scale bar: 10 µm. While glutamate appears to induce a directional anterograde movement, exogenous NAD preferentially promotes mitochondrial retrograde transport (quantified in (**E**,**F**)). Bars represent the mean ± SEM of five independent experiments. * represents *p* < 0.05 when compared to control. Statistical analysis was conducted with ANOVA test (Sidak’s multiple comparison test) and two-way ANOVA.

Taking into consideration the association between mitochondrial dynamics and transport, we next evaluated whether glutamate exposure affects mitochondrial movement and transport in neurites. Analysis of TMRM labeling in cortical neurons exposed to glutamate by live cell imaging revealed an increase in all mitochondria movement metrics. We observed an increase of around 17% in velocity and 12% in mitochondrial displacement that was reversed when neurons were pretreated with NAD (Figure 4A–C).

To further investigate mitochondrial mobility, we analyzed the directionality of mitochondrial movement in neurites (Figure 4D). Although in control neurons no differences were evident in the number of retrogradely vs. anterogradely moving mitochondria, with an anterograde/retrograde ratio of approximately 1, treatment with glutamate altered mitochondrial transport, as indicated by an increase of the ratio to 0.79 (Figure 4E). This directionality appears to result from decreased anterograde and retrograde movement; however, the number of mitochondria being transported toward the soma has a more pronounced reduction compared to control (Figure 4F). Exposure to NAD before glutamate stimulation recovers retrograde transport of mitochondria, with an anterograde/retrograde ratio nearing 1.62.

### 3.5. NAD and NAD Biosynthetic Pathway Precursors Preserve the Metabolic Functions of Neurons Against Glutamate Excitotoxicity

**Figure 5:**
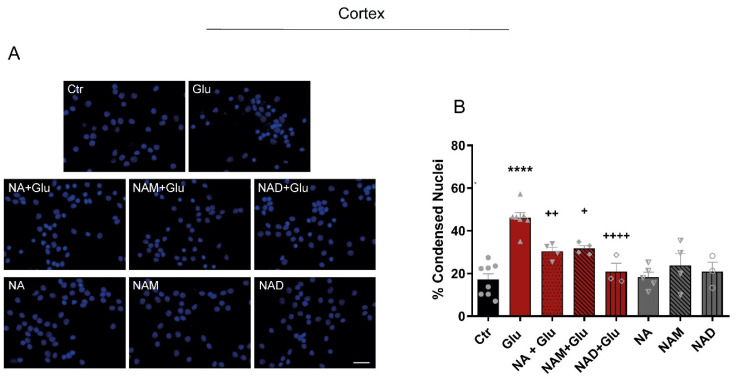
Stimulation with NAD or NAD biosynthetic pathway precursors protects against glutamate-induced excitotoxicity. (**A**) Representative images of Hoechst staining of cortical neurons. Cortical neurons were exposed to 50 µM glutamate in the presence or absence of NAD or its precursors. Cell death was evaluated by counting the number of condensed nuclei stained with the fluorescent dye Hoechst 33342. Scale bar: 30 µm. (**B**) Quantitative data of cell death. Glutamate exposure increased the percentage of condensed nuclei in cortical neurons, which was reverted by NA, NAM, and NAD. Results are expressed as a percentage of control. Control (circles); Glutamate (triangles); Glutamate + NA (upside down triangle); Glutamate + NAM (filled diamonds); Glutamate + NAD (Unfilled diamonds); NA(triangle filled only on the left side); NAM (triangle filled only on the right side); NAD(empty circle) Bars represent the mean ± SEM of three independent experiments. Statistical significance was assessed by ANOVA followed by the Bonferroni multiple comparison test. **** represents *p* < 0.0001 when compared to control. + represents *p* < 0.05; ++ represents, and ++++ represents *p* < 0.0001 when compared to glutamate.

To evaluate the effect of NAD metabolism in glutamate-induced excitotoxicity, NAD and NAD biosynthesis precursors were applied alone or in conjunction with 50 µM glutamate to cortical neurons. Cell death was determined by analyzing nuclear condensation using Hoechst 33342 (Figure 5A). Cortical neurons under control conditions showed an absolute cell death of 17.15%; in turn, glutamate increased cell death to 46.11%. This effect was reverted when NA (30.34%), NAM (31.71%), and NAD (20.92%) were added prior to the glutamate insult (Figure 5B).

**Figure 6:**
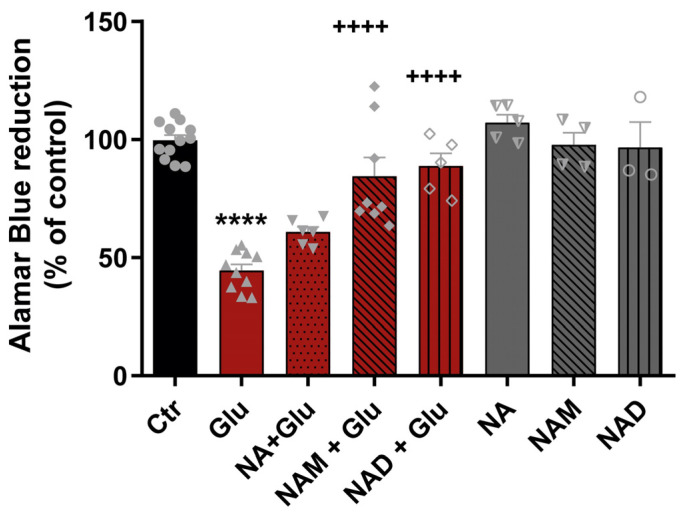
Stimulation with NAD or NAD biosynthetic pathway precursors protects against glutamate-induced metabolic dysfunction. Cortical neurons were exposed to 50 µM glutamate in the presence or absence of NAD or its precursors, and cell metabolic activity was measured by the Alamar Blue assay. Glutamate decreased cell metabolic activity, which was reverted by NAD or its precursors in both types of neurons. Control (circles); Glutamate (triangles); Glutamate + NA (upside down triangle); Glutamate + NAM (filled diamonds); Glutamate + NAD (Unfilled diamonds); NA(triangle filled only on the left side); NAM (triangle filled only on the right side); NAD(empty circle) Statistical significance was assessed by ANOVA followed by the Bonferroni multiple comparison test. **** represents *p* < 0.0001 when compared to control. ++++ represents *p* < 0.0001 when compared to glutamate.

We then sought to investigate whether the NAD biosynthetic pathways were able to preserve the metabolic activity of CNS neurons against a glutamate insult. Cortical neurons were exposed to glutamate as described previously, and the metabolic function of neurons was assessed by the Alamar Blue assay. Glutamate induced a reduction in the metabolic activity of cortical neurons to 44.6% of control. However, when pre-incubated prior to glutamate, NAM and NAD rescued the decrease in cell metabolic activity induced by glutamate, to 84.48% and 88.77%, respectively. NA had no significant protection, but the results show an increase in metabolic activity to 60.89% when compared to glutamate alone (Figure 6). Overall, the results show that NAD and its precursors protect against glutamate-induced metabolic demise.

## 4. Discussion

Excitotoxicity stems from excessive release of glutamate receptors residing in the postsynaptic neuron, most notably the NMDAR, which results in cell death. The multifactorial nature of excitotoxicity combines calcium overload, mitochondrial impairment, oxidative stress, and failure in energy metabolism, mainly due to NAD depletion. We observed that NAD biosynthetic pathways are altered in CNS neurons in response to a glutamate insult. Surprisingly, no alterations in NAPRT expression were observed (Figure 1), despite NA protection against glutamate damage (Figure 6). Two recent studies emphasized a neuroprotective effect for NA (or nicotinic acid) in Alzheimer’s disease mouse models through the HCAR2 receptor activation in the microglia, supporting its anti-inflammatory role [18,19]. Future work may unveil the function of NA in the context of excitotoxicity. In turn, as expected, expression of the rate-limiting enzyme of the main route for NAD biosynthesis in mammals, NAMPT, increases after exposure to glutamate, which suggests a feedback response to NAD depletion. The subsequent and final step of the NAM pathway involves the action of NMNATs. These have distinct subcellular localizations, and we observed that the genes encoding nucleoplasmic NMNAT1 and cytosolic NMNAT2 increase early after glutamate exposure. Inversely, mitochondrial NMNAT3 decreased 24 h after glutamate exposure, which suggests that mitochondrial NAD levels also decrease and may play an important role in glutamate-induced excitotoxicity. The observed decrease in NMNAT3 may lead to a reduction in mitochondrial NAD levels, but additional factors must be taken into consideration. For example, the mitochondrial permeability transition pore (mPTP) is regulated by both mitochondrial and cytosolic NAD levels. During glutamate excitotoxicity, the calcium overload may trigger mPTP opening, contributing to the release of NADH from mitochondria and mitochondrial membrane potential depolarization, ultimately leading to neuronal cell death [20]. In terms of NAD glycohydrolases, the activity of CD38 was found to be higher in ischemic conditions [21]. Furthermore, CD38, positioned in the nuclear or mitochondrial membrane, can access intracellular NAD, and its activation may cause a substantial depletion of cellular NAD [22]. Increased cytosolic NAD biosynthesis and transport into mitochondria may provide a mechanism to compensate for these effects.

Using a fluorescent NAD sensor capable of monitoring NAD levels in the mitochondria, we observed that fluorescence increases after glutamate exposure (Figure 2), and these results indicate a depletion in the mitochondrial NAD pool. There is evidence that neuronal mitochondria possess an intact NAD salvage pathway, due to the expression of NAMPT and NMNAT3 enzymes and because knockdown of both NMNAT3 and NAMPT reduced ATP production-related respiration [23] and mitochondrial NAD levels [16]. Alternatively, some authors defend the existence of an NAD transporter capable of importing NAD from the cytosol to the mitochondria [24,25,26], while others state that NMN enters the mitochondria and is converted to NAD by the action of NMNAT3 enzyme [27]. The increase in NMNAT1 and NMNAT2 expression levels and the decrease in NMNAT3 following glutamate excitotoxicity suggested that the first scenario might occur, and our results sustain this argument since NAD addition could rescue the decrease in mitochondrial NAD levels observed after glutamate exposure. This result indicates that NAD protects mitochondria against glutamate-induced excitotoxicity, which should be further investigated. Recently, SLC25A51 (MCART1) was identified as a new mitochondrial NAD transporter in mammalian cells [25,26]. Future work may assess SLC25A51 expression/activation in our conditions and also whether NAD-mediated protection against glutamate damage involves NAD import to the mitochondria through this transporter.

Our results for NMNAT3 (mitochondrial isoform) are different from other studies that used NMDA as an excitotoxic agent and also from models of ischemia. NMNAT3 increased after NMDA stimulation in cortical neurons, and its knockdown in cortical neurons was associated with an increase in neuronal degeneration and excitotoxic cell death [10,28]. Furthermore, NMNAT3 was protective against the effects of neonatal cerebral hypoxia-ischemia [28]. Given these differences, future studies are needed to unveil the significance of NMNAT3 decrease in glutamate-induced excitotoxicity and the cellular and molecular mechanisms related to NMNAT3 knockdown in the model used in this study and other models of excitotoxicity and ischemia. Moreover, NAD availability in cell compartments such as the nucleus and the cytoplasm could be measured, as these can influence the mitochondrial NAD pool in the axon, as seen in other cellular models [16,17]. Overall, our results demonstrate that glutamate-induced excitotoxicity acts by compromising the NAD biosynthetic pathway, particularly in the mitochondrial NAD pool. The dynamics of mitochondria, movement, and the mitochondrial membrane potential are also altered by glutamate exposure (Figure 3 and Figure 4). Such change in mitochondrial mobility could be due to several different factors, such as increased recruitment at specific cell locations, impaired arresting processes, or dysregulation of transport mechanisms. The retrograde movement of mitochondria is partially reverted when exogenous NAD is added. Supplementation with extracellular NAD or NAD precursors can result in increased intracellular NAD concentrations. The direct uptake of extracellular NAD through the plasma membrane can counteract NAD depletion and enhance cell survival under stress conditions [29]. Alternatively, NAD can be degraded by ectoenzymes to NAM or NMN, which are then imported and used by NAD salvage enzymes, such as NAMPT and NMNATs [30] The increased expression levels of NAMPT, NMNAT1, and NMNAT2 observed in our model suggest that this mechanism may be used to regenerate the NAD pool, protecting cells from excitotoxicity.

We also observed that pre-incubation with NAD and its precursors was able to inhibit glutamate-induced cell death in cortical neurons (Figure 5).

Previous studies showed that NAD, when added together with glutamate, resulted in neuroprotection in high doses [11,31]. NAD and NR were not able to inhibit NMDA-induced nuclear condensation after 24 h of NMDA stimulation but instead protected against excitotoxicity-mediated axonal degeneration [10]. These observations indicate that NAD and/or NAD biosynthetic precursor availability need to be elevated prior to an excitotoxic challenge to confer somatic neuroprotection, and that the axon may be the first target of NAD-mediated neuroprotection against excitotoxicity.

Cell viability was also measured by assessing cell metabolic rate, and the results were in accordance with those obtained for cell death (Figure 6). It is important to mention that the Alamar Blue metabolic rate assay uses NADH as a reducing agent so that the NAD generated by the NAD biosynthetic precursors added could influence the cell metabolic rate results. However, similar outcomes were obtained when compared to the cell death assay, which reinforces the positive impact of NAD metabolism in the protection against excitotoxic damage. Overall, these results open the exciting possibility that the pool of mitochondrial NAD may be used as a tool for CNS regenerative therapies.

## Data Availability

The raw data supporting the conclusions of this article will be made available by the authors upon request.

## Supplementary Materials

The following supporting information can be downloaded at: https://www.mdpi.com/article/10.3390/cells14080582/s1, Figure S1: Co-localization of mitochondrial NAD sensor with TOM20; Figure S2: cpVenus control sensor shows no alterations upon glutamate or NAD stimulation; Table S1: Primers used to amplify the genes of interest in this study; Video S1: representative video of TMRM signaling in live cell.
