# Supplementary material for: Neuroprotection by Mitochondrial NAD Against Glutamate-Induced Excitotoxicity
Source: Cells. 2025 Apr 12;14(8):582. doi: 10.3390/cells14080582 (PMC12025592; doi:10.3390/cells14080582)
Supplement: Supplementary file 1 [file cells-14-00582-s001.zip › cells-3556205-supplementar.docx]

**Supplementary Figures**


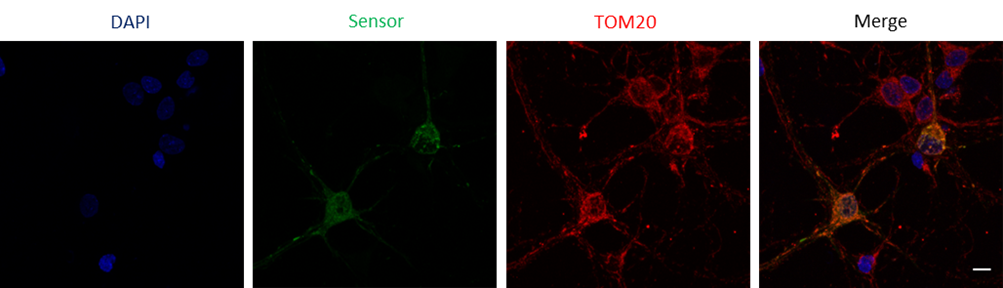


**Supplementary Figure 1 - Co-localization of mitochondrial NAD sensor with TOM20.**

Cortical neurons were transfected with the mitochondrial NAD sensor (green) and immunolabeled with an antibody against the mitochondrial marker TOM20 (red). Maximum intensity projections of Z-stacks were generated to obtain a single representative image. The results show that the NAD mitochondrial sensor co-localizes with TOM20 as expected. Scale bar: 10 µm.


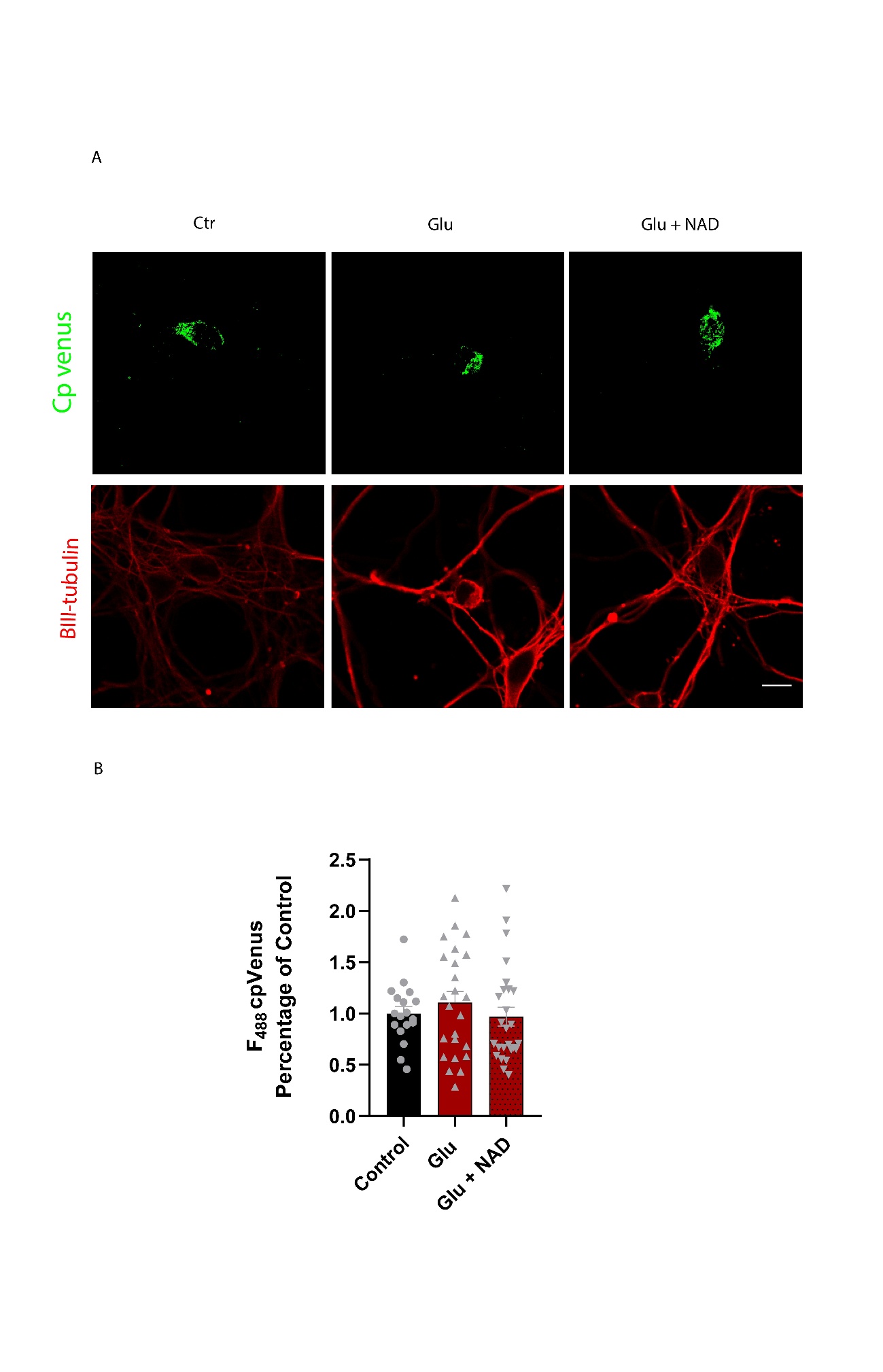


**Supplementary Figure 2 – cpVenus control sensor shows no alterations upon glutamate or NAD stimulation.**

1. Representative microscopy images of cortical neurons incubated with control sensor and βIII-tubulin**.** Cortical neurons were transfected with 3 µM the control NAD sensor cpVenus (green) and immunolabeled with an antibody against the neuronal marker βIII-tubulin (red) (A). Maximum intensity projections of Z‐stacks were assembled to generate a single image. Scale bar: 10 µm.No alterations were detected in fluorescence intensity in all the conditions tested **(B)** Quantitative data of control sensor fluorescence. Bars represent the mean ± SEM of at least 20 neurons randomly selected from 3 independent experiments. Statistical significance was assessed by ANOVA followed by the Bonferroni multiple comparison test. No statistical difference was observed between conditions.

**Supplementary Table 1: Primers used to amplify the genes of interest in this study.**

| **Gene** | **Forward Primer sequence**  **5´- 3´** | **Reverse Primer sequence**  **5´- 3´** | **Length (bp)** | **Ta (ºC)** |
| --- | --- | --- | --- | --- |
| *NAMPT* | GGCTACAGAGGAGTCTCTTC | GCTGGAACAGAATAGCCTGG | 151 | 57 |
| *NMNAT1* | TCACCAACATGCACCTCAGG | TGGCAAGTTCCGCCATGATG | 162 | 57 |
| *NMNAT2* | ACCGTCTCATCATGTGTCAGC | GATCACAGGTGTCATGGAAGG | 185 | 57 |
| *NMNAT3* | CACATCCAGGAGATAGTGGAG | GATGTATGTGGCACTGATCTC | 162 | 57 |
| *18s* | GTTCCGACCATAAACGATGCC | TGGTGGTGCCCTTCCGTCAAT | 170 | 57 |

Ta: Annealing temperature
